# Oxidative stress and apoptosis induction in human thyroid carcinoma cells exposed to the essential oil from *Pistacia lentiscus* aerial parts

**DOI:** 10.1371/journal.pone.0172138

**Published:** 2017-02-14

**Authors:** Simona Catalani, Francesco Palma, Serafina Battistelli, Serena Benedetti

**Affiliations:** 1 Department of Biomolecular Sciences, Section of Clinical Biochemistry and Molecular Genetics, University of Urbino “Carlo Bo”, Urbino, Italy; 2 Department of Biomolecular Sciences, Section of Biochemistry and Molecular Biology, University of Urbino “Carlo Bo”, Urbino, Italy; Wayne State University, UNITED STATES

## Abstract

**Background:**

Essential oils from the aerial parts (leaves, twigs and berries) of *Pistacia lentiscus* (PLEO) have been well characterized for their antibacterial and anti-inflammatory properties; however, poor information exists on their potential anticancer activity.

**Methods:**

Increasing concentrations of PLEO (0.01–0.1% v/v, 80–800 μg/ml) were administered to a wide variety of cultured cancer cells from breast, cervix, colon, liver, lung, prostate, and thyroid carcinomas. Fibroblasts were also included as healthy control cells. Cell viability was monitored by WST-8 assay up to 72 hours after PLEO administration. The intracellular formation of reactive oxygen species (ROS), the induction of apoptosis, and the enhancement of chemotherapeutic drug cytotoxicity by PLEO were further investigated in the most responsive cancer cell line.

**Results:**

A dose-dependent reduction of tumor cell viability was observed upon PLEO exposure; while no cytotoxic effect was revealed in healthy fibroblasts. FTC-133 thyroid cancer cells were found to be the most sensitive cells to PLEO treatment; accordingly, an intracellular accumulation of ROS and an activation of both the extrinsic and intrinsic apoptotic pathways were evidenced in FTC-133 cells after PLEO administration. Furthermore, the cytotoxic effect of the antineoplastic drugs cisplatin, 5-fluorouracil and etoposide was enhanced in PLEO-exposed FTC-133 cells.

**Conclusion:**

Taking into account its mode of action, PLEO might be considered as a promising source of natural antitumor agents which might have therapeutic potential in integrated oncology.

## Introduction

Essential oils (EOs) are natural, volatile, and odorous molecules synthesized by the secretory cells of aromatic plants, located in leaves, flowers, fruits, seeds, barks, and roots [1]. Mainly composed of terpenes and terpenoids [2], EOs are currently receiving therapeutic interest fully renewed not only for their well-documented antimicrobial [3, 4], antioxidant [5] and anti-inflammatory [6] activities, but also for their anticancer properties [7, 8] and synergist effect with conventional therapies [9, 10].

Among EO-bearing plants, *Pistacia lentiscus* L. (PL), an evergreen bush of the Anacardiaceae family that extensively thrives in the Mediterranean area, has attracted considerable attention for its wide variety of bioactivities [11, 12]. In particular, an increasing number of studies has revealed that PL trunk resin (namely mastic gum) may exert anticancer activity in several types of human neoplasia, including prostate, colon, lung, and pancreatic carcinomas as well as hematological malignancies [13–15].

Other than from mastic gum, EOs can be extracted from PL aerial parts such as leaves, twigs, flowers, and berries. However, while antibacterial and anti-inflammatory properties have been widely demonstrated for EOs from PL aerial parts [16, 17], poor information exists on their potential anticancer activity. In the present paper, we reported for the first time on the antiproliferative effects of an EO extracted from PL aerial parts on different cultured cancer cells, demonstrating its capability to reduce tumor cell viability through the intracellular accumulation of reactive oxygen species (ROS) and the induction of apoptotic cell death.

## Methods

### *Pistacia Lentiscus* Essential Oil (PLEO)

PLEO, extracted from leaves, twigs and berries of PL from Sardinia (Italy), was produced by SSA Mediflora (Cagliari, Italy). Its chemical composition is shown in Table 1. The oil was kept in the dark at room temperature; immediately before use, a stock containing 1% PLEO (solubilized in the culture medium containing 1% dimethyl sulfoxide, DMSO) was prepared and sterilized using 0.45 μm filters. The same lot was used for all the experiments on cultured cells. The following PLEO concentrations were tested: 0.01, 0.02, 0.04, 0.06, 0.08, and 0.1% (v/v), corresponding to 80, 160, 320, 480, 640, and 800 μg/ml, respectively. PLEO working dilutions contained up to 0.1% DMSO, thus avoiding solvent toxicity.

**Table 1:** Chemical composition of the EO extracted from PL aerial parts.^a^

Compound	%
Myrcene	25,25
alpha-Pinene	18,64
Limonene + beta-Phellandrene	9,77
alpha-Phellandrene	7,31
4-Terpineol	7,09
gamma-Terpinene	3,48
beta-Pinene	3,11
Sabinene	2,47
alpha-Terpinene	2,43
Germacrene D	2,04
p-Cymene	1,69
alpha-Terpineol	1,58
delta-Cadinene	1,51
Canphene	1,34
Terpinolene	1,21
beta-Caryophyllene	1,18
trans-beta-Ocimene	0,91
gamma-Muurolene	0,51
alpha-Thujene	0,5
Bornyl acetate	0,41
alpha-Muurolene	0,37
2-Undecanone	0,35
alpha-Caryophyllene	0,35
Tricyclene	0,33
alpha-p-Dimethylstyrene + 2-Nonanone	0,31
Nonanal	0,3
tau-Muurolol	0,28
cis-beta-Ocimene	0,24
gamma-Cadinene	0,21
alpha-Cadinol	0,21
alpha-Copaene	0,2
Isoamyl butyrate	0,18
beta-Elemene	0,17
Aromadendrene	0,16
beta-Bisabolene	0,15
2-Nonanol	0,14
Borneol	0,12
gamma-Terpineol	0,08
Sabinene hydrate	0,07
Isoamyl hexanoate	0,07
2-Methylbutyl hexanoate	0,07
alpha-Cubebene	0,07
alpha-Terpinyl acetate	0,06
Amyl benzoate	0,06
Calacorene + alpha-Bisabolene	0,06
delta-Cadinol	0,06
beta-Bourbonene	0,05
beta-Cubebene	0,04
2-Undecanol	0,02
Others	2,79

^a^Analysis were carried out by the laboratory Chelab Silliker (Treviso, Italy) by gas chromatography/mass spectrometry (GC/MS) and gas chromatography/flame ionization detection (GC/FID). Chromatographic area percentages (%) of the compounds are represented.

### Cell culture

The following cancer cell lines were employed: CaCo-2 (colon adenocarcinoma), FTC-133 (follicular thyroid carcinoma), HeLa (cervix carcinoma), Hep G2 (liver carcinoma), LNCaP (prostate carcinoma), MDA-MB-231 (breast carcinoma), and NCI-H1975 (non-small cell lung adenocarcinoma). Fibroblasts (AG-09429) were used as healthy control cells. All cell lines were available at the Department of Biomolecular Sciences, University of Urbino “Carlo Bo”, with the exception of FTC-133, Hep G2, and NCI-H1975 cells, which were obtained from Interlab Cell Line Collection (ICLC, Genova, Italy). HeLa, LNCaP, and NCI-H1975 cells were grown in RPMI 1640 medium supplemented with 10% fetal bovine serum (FBS), 1% L-glutamine, and 1% penicillin/streptomycin 100 U/ml. AG-09429 and FTC-133 cells were grown in DMEM medium supplemented with 10% FBS, 1% L-glutamine, and 1% penicillin/streptomycin 100 U/ml; CaCo-2, Hep G2, and MDA-MB-231 were grown in the same conditions but with the addition of 1% non-essential amino acids. All cells were maintained in a CO_2_ incubator at 37°C and 5% CO_2_. Cell culture materials and reagents were from VWR International (Milan, Italy).

### Cell viability assay

Cell viability upon PLEO administration was analyzed at 450 nm by the WST-8 reagent [2-(2-methoxy-4-nitrophenyl)-3-(4-nitrophenyl)-5-(2,4-disulfophenyl)-2H-tetrazolium, monosodium salt] (Sigma-Aldrich, Milan, Italy). The assay was based on the cleavage of the tetrazolium salt WST-8 by cellular dehydrogenases in viable cells. Briefly, cancer and healthy cell lines (1000–5000 cells/well) were incubated in 96-well plates with PLEO (0.01–0.1% v/v, 80–800 μg/ml) or vehicle (0.1% DMSO, untreated controls). After 24, 48, and 72 hours of incubation, WST-8 (1:10 final dilution) was added to each well, and cells were further incubated at 37°C up to 4 hours. Color development was monitored at 450 nm in a multiwell plate reader (Thermo Fisher Scientific, Milan, Italy). Data were plotted and IC_50_ values (i.e. PLEO concentration required to reduce cell viability by 50%) were then calculated. In FTC-133 cells, cell viability after PLEO administration was evaluated even in the presence of the antioxidant glutathione (GSH, final concentration 5 mM) (Sigma-Aldrich, Milan, Italy).

### ROS evaluation

Intracellular ROS formation upon PLEO administration to cancer and healthy cell lines was analyzed by the use of 2′,7′-dichlorofluorescin diacetate (DCFH-DA) (Sigma-Aldrich, Milan, Italy) [18]. DCFH-DA is a cell-permeable non-fluorescent probe which turns to highly fluorescent 2′,7′-dichlorofluorescein upon oxidation. Briefly, cells (5000/well) were incubated in black 96-well plates with PLEO (0.01–0.08% v/v, 80–640 μg/ml) or vehicle (0.1% DMSO, untreated controls) for 6 hours. DCFH-DA (final concentration 5 μM) was added to each well for 30 min; after excess probe removal, fluorescence was monitored at Ex/Em 485/520 nm in the multiwell plate reader FluoStar Optima (BMG Labtech, Germany). In FTC-133 cells, intracellular ROS formation after PLEO administration was evaluated even in the presence of GSH (final concentration 5 mM).

### Apoptosis evaluation

Apoptosis induction by PLEO was investigated in FTC-133 cells by monitoring both caspase-8 and caspase-9 activity (the main effectors of the extrinsic and intrinsic apoptotic pathways, respectively), as well as DNA fragmentation (a common marker of late apoptosis).

Briefly, caspase-8 and -9 activation was determined in cell lysates after 6 and 24 hours upon PLEO administration (0.04% v/v, 320 μg/ml) using two colorimetric kits from Biovision (Milpitas, CA, USA) in accordance with the manufacturer’s instructions. Assays were based on the spectrophotometric detection at 405 nm of the chromophore *p*-nitroaniline (*p*NA) after cleavage from the labeled substrate ETD-*p*NA by caspase-8 or LEHD-*p*NA by caspase-9. Protein concentration in the cytosolic extracts was measured using the Bradford method [19].

Genomic DNA fragmentation was evaluated after 6, 24 and 48 hours upon PLEO administration (0.04% v/v, 320 μg/ml) by agarose gel electrophoresis, as previously described [20]. DNA samples were carefully resuspended in TE buffer; nucleic acid concentration and purity were measured using a NanoDrop® ND-1000 spectrophotometer (Thermo-Scientific, Wilminton, DE, USA). 2 μg of each sample was loaded onto 1.5% TAE agarose gel; DNA laddering was visualized on a UV transilluminator by ethidium bromide staining. Images were obtained using a Gel Doc 2000 (Bio-Rad Laboratories S.r.l, Segrate, MI, Italy).

### Mitochondrial Membrane Potential (MMP) evaluation

MMP was determined in FTC-133 cells after 2, 4, and 6 hours upon PLEO administration (0.04–0.08% v/v, 320–640 μg/ml) using a fluorometric kit from Biovision (Milpitas, CA, USA). The kit uses TMRE (tetramethylrhodamine, ethyl ester) to label active mitochondria. TMRE is a cell permeant, positively-charged, red-orange dye that readily accumulates in active mitochondria due to their relative negative charge. Depolarized or inactive mitochondria have decreased membrane potential and fail to sequester TMRE. Fluorescence was monitored at Ex/Em 549/575 nm in the multiwell plate reader FluoStar Optima (BMG Labtech, Germany).

### Hypoxia-Inducible Factor-1 alpha (HIF-1α) measurement

HIF-1α quantification was performed in FTC-133 cells upon PLEO administration (0.04% v/v, 320 μg/ml) using an enzyme-linked immunosorbent assay kit from Abcam (Cambridge, UK), in accordance with the manufacturer’s instructions. Color development was evaluated at 450 nm in a multiwell plate reader (Thermo Fisher Scientific, Milan, Italy). Protein concentration in cell extracts was measured using the Bradford method [19].

### Clonogenic assay

The clonogenic cell survival assay determines the ability of a cell to proliferate indefinitely, thereby retaining its reproductive ability to form a large colony or a clone. To test PLEO effects on FTC-133 colony formation capacity, cells (40000/cm^2^) were pretreated for 24 hours with increasing concentrations of PLEO (0.04–0.08% v/v, 320–640 μg/ml). Cells were harvested, 1000 viable cells were plated in 6-well plates and allowed to grow for 14 days. Colonies were then stained for 90 min at room temperature with 0.25% methylene blue in 50% ethanol; pictures were captured digitally and analyzed using a software for densitometric analysis (Quantity One 4.0.1, Bio-Rad Laboratories, Milan, Italy) to evaluate the colony volumes. Data were expressed as percentage of inhibition of colony formation compared to the control.

### Co-treatment with cisplatin, 5-fluorouracil, and etoposide

To evaluate PLEO ability to enhance the cytotoxicity of conventional chemotherapeutic drugs, FTC-133 cells were treated up to 48 hours with cisplatin (CDDP, 5–80 μM), 5-fluorouracil (5-FU, 25–500 μM), or etoposide (VP16, 5–100 μM) in the presence or absence of PLEO (0.04% v/v, 320 μg/ml). Cell viability was analyzed at 450 nm by the WST-8 reagent. Drugs were from Sigma-Aldrich (Milan, Italy).

### Statistical analysis

Data are presented as mean ± standard deviation (SD) of at least three independent experiments and analyzed by Student’s t-test to compare treated vs. untreated cells. Significance level was set at p<0.05 for all analysis.

## Results

### Cell viability inhibition and intracellular ROS formation by PLEO

**Fig 1:**
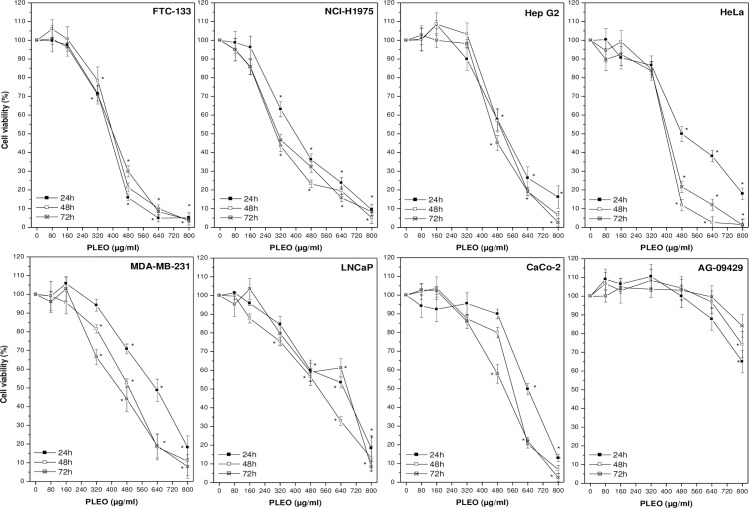
Cell viability evaluation by WST-8 colorimetric assay upon PLEO administration (0.01–0.1% v/v, 80–800 μg/ml) to cancer and healthy cell lines for 24, 48, and 72 h. Data are expressed as mean ± SD (n = 3). *p<0.05 vs. untreated cells.

PLEO was administered to cancer and healthy cell lines up to 72 hours at concentrations ranging from 0.01 to 0.1% v/v (80–800 μg/ml). Overall, a dose-dependent inhibition of cancer cell viability by PL was observed as compared to untreated cells receiving the vehicle (0.1% DMSO) (Fig 1). In most cases, the maximum concentration tested (0.1% v/v, 800 μg/ml) was cytotoxic, leading to the detection of necrotic cells. As reported in Table 2, the most sensitive cancer cell line was FTC-133 (follicular thyroid carcinoma), showing the lowest IC_50_ value (376±20 μg/ml) after 24 hours of incubation with PL; on the contrary, AG-09429 healthy fibroblasts were the most resistant cells to PL treatment (IC_50_ >800 μg/ml).

**Table 2:** Antiproliferative activity of PLEO at 24, 48, and 72 h of treatment expressed as IC_50_ values (mean ± SD of three independent experiments).^a^

	IC_50_ (μg/ml)		
Cell lines	24 h	48 h	72 h
FTC-133	376±20	392±30	408±83
NCI-H1975	400±52	304±27	336±37
Hep G2	512±70	496±69	472±85
HeLa	520±42	392±27	408±45
MDA-MB-231	616±76	480±13	432±33
LNCaP	616±19	520±75	624±21
CaCo-2	640±13	552±13	520±45
AG-09429	>800	>800	>800

^a^IC_50_: PLEO concentration required to reduce cell viability by 50% in WST-8 assay.

**Fig 2:**
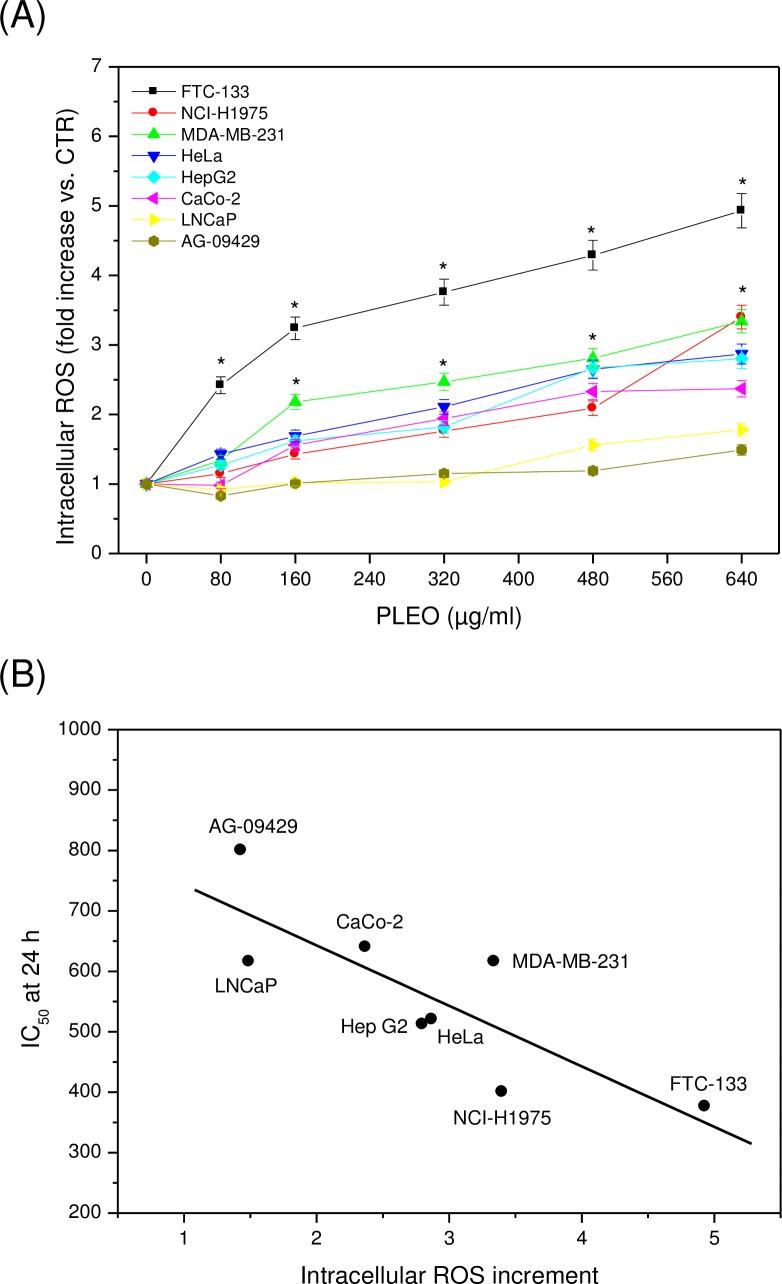
(A) Intracellular ROS increment after 6 h of PLEO administration (0.01–0.08% v/v, 80–640 μg/ml) to cancer and healthy cell lines. Data are expressed as mean ± SD (n = 3). *p<0.05 vs. untreated cells. (B) Negative correlation (R = -0.820, p = 0.013) between intracellular ROS increment after 6h-incubation of cancer and healthy cell lines with PLEO and IC_50_ values after 24 h of treatment. ROS increment values referred to those obtained incubating cells with the highest PLEO concentration tested (0.08% v/v, 640 μg/ml).

The administration of PLEO (0.01–0.08% v/v, 80–640 μg/ml) to cancer cell lines also led to a significant increment of intracellular ROS levels as compared to untreated cells receiving the vehicle (0.1% DMSO) (Fig 2A). FTC-133 cells showed the highest ROS content, while AG-09429 healthy cells the lowest. A significant negative correlation (R = -0.820, p = 0.013) was observed between ROS increment at 6 hours upon PL administration and IC_50_ values at 24 hours of treatment (Fig 2B).

**Fig 3:**
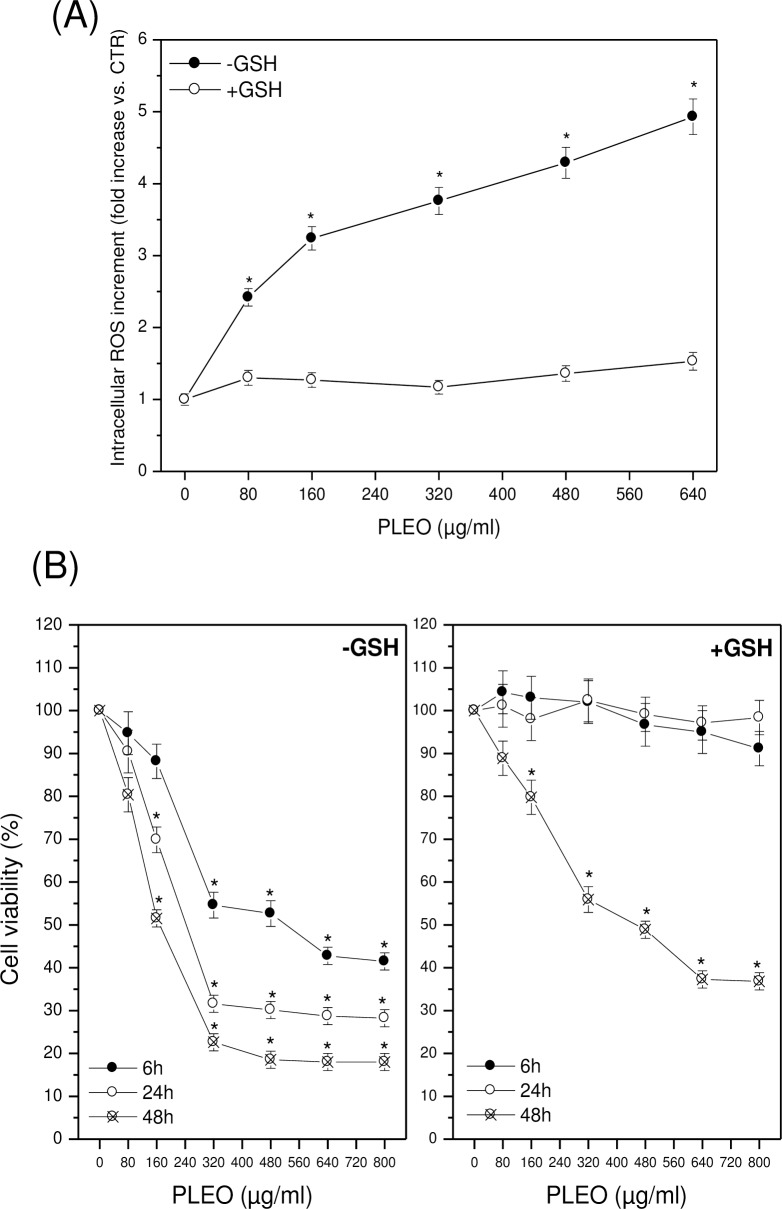
(A) Intracellular ROS levels after 6 h of PLEO administration (0.01–0.08% v/v, 80–640 μg/ml) to FTC-133 cells in the presence of GSH 5 mM. (B) Cell viability after 6, 24, and 48 h of PLEO administration to FTC-133 cells in the presence of GSH 5 mM. Data are expressed as mean ± SD (n = 3). *p<0.05 vs. untreated cells.

In FTC-133 cells, experiments were repeated in the presence of GSH (5 mM). No intracellular ROS accumulation was evidenced after 6 hours of cell incubation with increasing concentrations of PLEO (Fig 3A); at the same time, no inhibition of cell viability was found up to 24 hours after PL administration (Fig 3B). The protective effect of GSH decreased over time, leading to a significantly reduced cancer cell viability after 48 hours of treatment with PLEO.

### Apoptosis induction by PLEO

**Fig 4:**
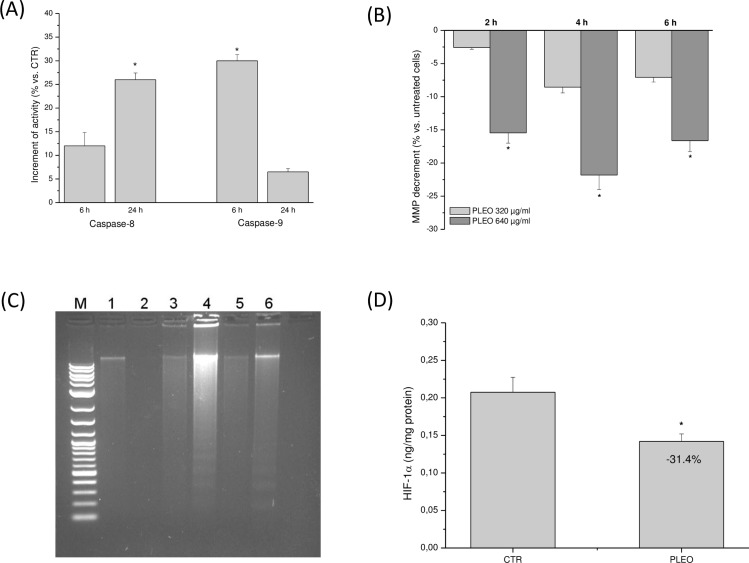
(A) Caspase-8 and -9 activation after 6 and 24 h of PLEO administration (0.04% v/v, 320 μg/ml) to FTC-133 cells. (B) MMP reduction in FTC-133 cells incubated for 2, 4, and 6 h with PLEO (0.04–0.08% v/v, 320–640 μg/ml). (C) DNA fragmentation analysis in FTC-133 cells after 6, 24, and 48 h upon PL treatment (0.04% v/v, 320 μg/ml). M: molecular marker; 1–2: 6 h-CTR and PLEO-treated cells; 3–4: 24 h-CTR and PLEO-treated cells; 5–6: 48 h-CTR and PLEO-treated cells. (D) Reduction of HIF-1α levels after 24 h upon PLEO administration (0.04% v/v, 320 μg/ml) to FTC-133 cells. Data are expressed as mean ± SD (n = 3). *p<0.05 vs. untreated cells.

The administration of PLEO (0.04% v/v, 320 μg/ml) to FTC-133 cells led to the activation of both the extrinsic and intrinsic apoptotic pathways. As shown in Fig 4A, a significant caspase-8 activation was observed after 24 hours upon PL administration; while caspase-9 activation was evidenced mostly after 6 hours of treatment. In accord to the early intrinsic pathway induction, a significant reduction of MMP was found at 2, 4, and 6 hours of incubation with PLEO as compared to untreated cells (Fig 4B). The presence of DNA fragmentation after 24 and 48 hours upon PL treatment confirmed apoptosis induction by the EO in FTC-133 cells (Fig 4C). Interestingly, PLEO-induced apoptosis was associated with a significant decrement of the intracellular levels of HIF-1α (-31.4% vs. untreated cells) (Fig 4D).

### Colony formation inhibition by PLEO

**Fig 5:**
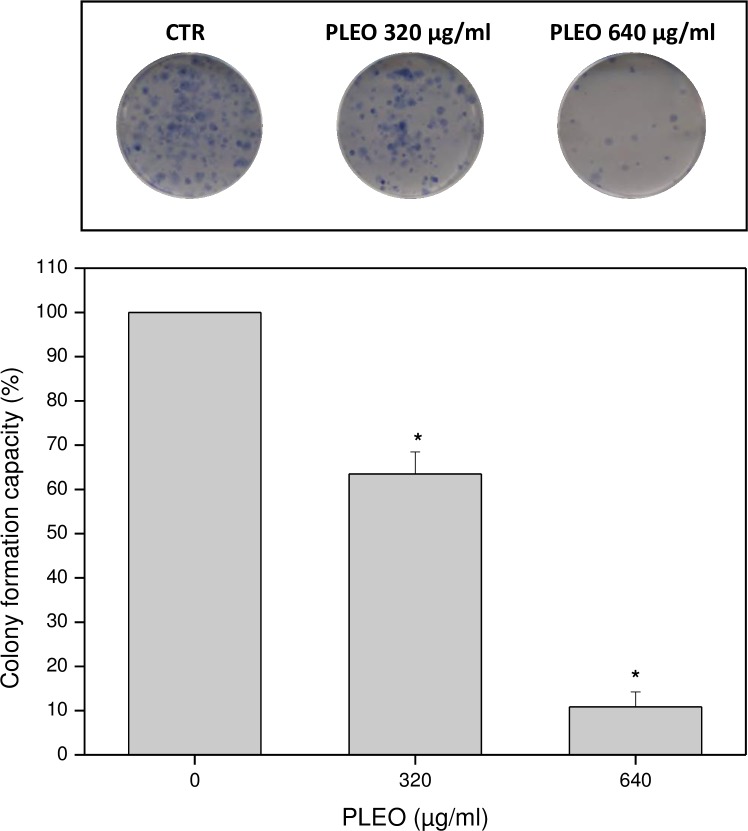
**Reduction of colony formation capacity after FTC-133 cell incubation with increasing PLEO concentrations (0.04–0.08% v/v, 320–640 μg/ml).** Data are expressed as mean ± SD (n = 3). *p<0.05 vs. untreated cells. The image is representative of three independent experiments.

As shown in Fig 5, a significant inhibition of FTC-133 colony formation capacity was observed upon PLEO administration (0.04–0.08% v/v, 320–640 μg/ml) to cancer cells in comparison to untreated cancer cells.

### Enhancement of chemotherapeutic drug cytotoxicity by PLEO

**Fig 6:**
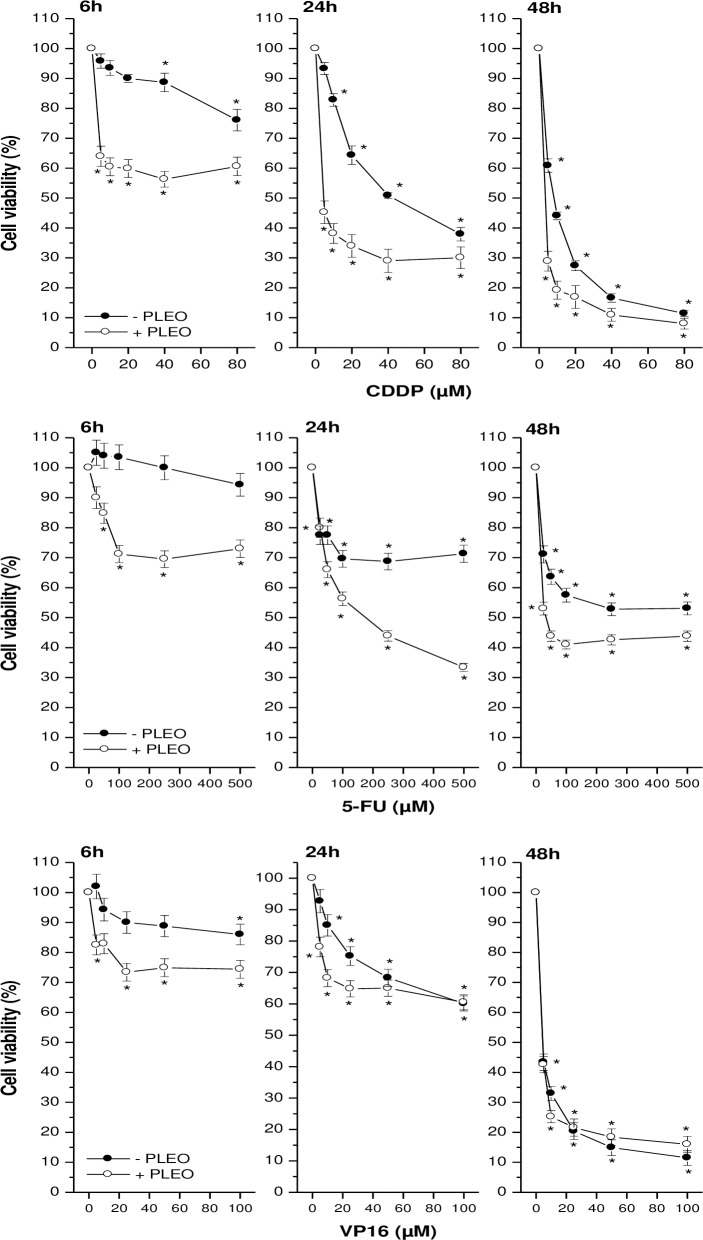
FTC-133 cell viability upon PLEO co-administration (0.04% v/v, 320 μg/ml) with increasing doses of CDDP (5–80 μM), 5-FU (25–500 μM), and VP16 (5–100 μM) for 6, 24, and 48 h. Data are expressed as mean ± SD (n = 3). *p<0.05 vs. untreated cells.

The antiproliferative effects of PLEO (0.04% v/v, 320 μg/ml) were determined in FTC-133 cancer cells also in presence of CDDP, 5-FU, and VP16. As shown in Fig 6, cell incubation with increasing concentrations of CDDP alone (5–80 μM) led to a significant decrement of cell viability as compared to untreated cells. The co-treatment with PL further decreased cell viability in particular after 24 hours upon administration (IC_50_ values equal to 43.5 μM and 4.2 μM in absence and presence of PLEO, respectively). As regards 5-FU, IC_50_ values were not reached up to 48 hours of incubation with the drug alone (25–500 μM); on the contrary, the co-treatment with PL allowed a decrease of cell viability so as to achieve the IC_50_ value both at 24 (172 μM) and 48 hours (33 μM). In the case of VP16 (5–100 μM), IC_50_ values, achieved only after 48 hours of incubation, were the same in absence or presence of PLEO (4.4 μM).

## Discussion

In the present study we investigated for the first time the antiproliferative properties of an essential oil extracted from the aerial parts of *Pistacia lentiscus* (PLEO) on a wide variety of cultured cancer cells from human breast, cervix, colon, liver, lung, prostate, and thyroid carcinomas. Fibroblasts were also included as healthy control cells.

Overall, a dose-dependent inhibition of cancer cell viability by PLEO was observed as compared to untreated cells. On the basis of IC_50_ values, FTC-133 (follicular thyroid carcinoma) was the most sensitive cancer cell line, while AG-09429 healthy fibroblasts were the most resistant cells to PL administration, thus evidencing a selective cytotoxic action of the EO against tumor cells. Other than cell viability, PLEO also inhibited the colony formation capacity of FTC-133 cells, proposing that some EO constituents might affect single tumor cell survival so as to suppress cancer cell colonization, as previously demonstrated [21].

On the basis of the chemical composition, the monoterpenes myrcene and α-pinene were the most abundant compounds of PLEO, suggesting that the antiproliferative effects of PL might be possibly mediated by these two compounds. In accord, previous evidence indicated that myrcene and α-pinene might exert significant cytotoxic effects on different cancer cell lines [22–24]. Nevertheless, the involvement of other PLEO minor constituents should not be ruled out; indeed, the terpenes limonene, β-caryophyllene, and β-elemene have also shown significant anticancer activities both *in vitro* and *in vivo* models [9], indicating potential synergies among EO components.

The administration of PLEO to cancer cell lines was also associated to a significant increment of intracellular ROS levels as compared to untreated cells. FTC-133 cells showed the highest increase, while AG-09429 healthy cells the lowest. Consequently, a significant negative correlation was observed between ROS increment and cell proliferation upon PL treatment. When experiments were repeated in the presence of GSH as antioxidant, no intracellular ROS accumulation and no inhibition of cancer cell growth were observed in FTC-133 cells after PL administration, thus indicating that PLEO might act as antiproliferative agent in a ROS-dependent manner. In accord, several terpenic EO constituents, such as α-pinene and β-caryophyllene, have demonstrated to specifically induce the production of ROS within cancer cells without increasing oxidative stress in normal cells [22, 25].

The specific action of PLEO towards cancer cells might be partially related even to cell mitotic rate. Indeed, FTC-133 cell line, the most sensitive to PLEO administration, also presented the lowest doubling time (approximately 27 hours) among the cell lines tested, possibly indicating that some PLEO components (such as limonene and β-elemene) might interfere with cell cycle and DNA synthesis [9, 23].

To clarify the mechanisms underlying PLEO antiproliferative effects, apoptosis was investigated in FTC-133 cells. We found that PLEO led to the activation of both the intrinsic and extrinsic apoptotic pathways, as revealed by caspase-9 activation after 6 hours of treatment and by caspase-8 activation after 24 hours upon PL administration. In accord to the early activation of the intrinsic pathway, a significant loss of mitochondrial membrane potential was found up to 6 hours of incubation with PLEO. The presence of nuclear DNA fragmentation after 24 and 48 hours upon PL treatment confirmed apoptosis induction by the EO in FTC-133 cells.

Interestingly, FTC-133 is a cancer cell line characterized by the mutation of the tumor suppressor gene PTEN (phosphatase and tensin homologue) [26], which makes the transcription factor HIF-1α functionally expressed in thyroid carcinomas independently of lowered oxygen tension, thus promoting apoptosis resistance and tumor cell survival [27]. In this context, we observed that PLEO-induced apoptosis in FTC-133 cells was associated with a significant decrement of HIF-1α levels, possibly indicating a negative modulation of the hypoxic factor by some PLEO components such as the sesquiterpene β-elemene, which has demonstrated to enhance radiosensitivity *in vivo* via HIF-1α downregulation [28].

Positively, we evidenced that PLEO enhanced the inhibitory effects of the chemotherapeutic drugs CDDP, 5-FU and VP16 on FTC-133 cell proliferation. This action was particularly effective in the case of 5-FU; indeed, PLEO co-administration favourably allowed the achievement of IC_50_ values, thus confirming the role of EO constituents in potentiating the anticancer capabilities of conventional chemotherapeutic agents [9].

In conclusion, this study provided new insights into the antitumor action of the essential oils from *Pistacia lentiscus* aerial parts, for which poor investigations exist. Being a complex mixture of numerous constituents, PLEO action on cancer cells via intracellular ROS accumulation and apoptosis induction might be the sum of each individual activity, modulated by all the potential interactions. Taking into account its mode of action and its ability to enhance the cytotoxic effect of conventional antineoplastic drugs, PLEO might be considered as a promising source of natural antitumor agents and might have therapeutic potential in integrated oncology as a support to the standard anticancer therapies.
